# Editorial: The legacy of Dr. Rita Levi-Montalcini: advances in neurotrophic factors in brain disease development and treatment

**DOI:** 10.3389/fnmol.2024.1387026

**Published:** 2024-04-25

**Authors:** Wei-Jung Chen, Woon-Man Kung, Muh-Shi Lin

**Affiliations:** ^1^Department of Biotechnology and Animal Science, College of Bioresources, National Ilan University, Yilan, Taiwan; ^2^Division of Neurosurgery, Department of Surgery, Taipei Tzu Chi Hospital, Buddhist Tzu Chi Medical Foundation, New Taipei City, Taiwan; ^3^Department of Exercise and Health Promotion, College of Kinesiology and Health, Chinese Culture University, Taipei, Taiwan; ^4^Department of Health Business Administration, College of Medical and Health Care, Hung Kuang University, Taichung, Taiwan; ^5^Division of Neurosurgery, Department of Surgery, Kuang Tien General Hospital, Taichung, Taiwan

**Keywords:** editorial, the legacy of Dr. Rita Levi-Montalcin, nerve growth factor (NGF), brain-derived neurotropic factor (BDNF), brain injuries and diseases

Rita Levi-Montalcini, originally from Italy and Jewish, discovered nerve growth factor (NGF), for which she was awarded the 1986 Nobel Prize in Physiology or Medicine along with her colleague Stanley Cohen. The groundbreaking discovery of NGF has made Dr. Rita Levi-Montalcini one of the most distinguished scientists and celebrities in the field of neuroscience. Throughout her life (1909–2012), Rita Levi-Montalcini endured persecution under Mussolini's fascist dictatorship and encountered gender and religious discrimination. However, these adversities contributed to her drive and resilience, which ultimately led to her being awarded the Nobel Prize. Regardless of these obstacles, she pursued her academic career diligently and gracefully, and exerted an immeasurable impact on the field of science. As a signaling protein, as well as an exogenous stimulator of neurons, NGF, has a number of target cells in the nervous system and impacts on the growth and differentiation of specific populations of neurons, which contributes to the mechanisms of cell growth and maintenance. The purpose of this Research Topic is to highlight and profile neurotrophic factors, as a valuable legacy of Dr. Levi-Montalcini's research, the latest advances and approaches in the development and treatment of brain disorders.

Within the brain, neuronal stability and overall coherence must be properly ensured, whilst internal support and immune capabilities are guaranteed by astrocytes, microglia (glial cells) and the blood-brain barrier (BBB). Under circumstances as traumatic brain injury, cerebrovascular accidents, and neurodegenerative diseases of the brain (hereinafter referred to generically as brain injuries and diseases), the self-fulfilling regions of the brain are implicated and interrupted. Namely, the double-edged sword of the neuroinflammatory response has no more preventive function for nerve regeneration, but is further overtaken by pro-inflammatory consequences in the brain. In neuroinflammatory conditions, alterations in BBB permeability are observed, restricting BBB from obstructing the entry of external substances, bacteria, or viruses across to the brain via the peripheral circulation (Lin et al., 2023). Concretely, when excessive neuroinflammatory reactions to a stressor emerge in the brain, astrocyte activation and subsequent reactive astrocyte proliferation may contribute to modifications in BBB permeability. Accordingly, as this defense between the peripheral system and the brain is undermined, a plurality of pro-inflammatory cytokines and chemokines released by neuroglial cell activation attracts peripheral innate immune cells, e.g., neutrophils, monocytes, macrophages, natural killer cells, and dendritic cells, toward the brain. A vicious circle emerges from the accumulation of innate and peripheral immune cells and the amplification of inflammatory signals by neuroglial cytokines/coagulation factors spreading throughout the neuroinflammatory process in the brain. This neuroinflammatory response magnifies and proceeds to mitochondrial dysfunction (Kung and Lin, 2021) in the principal neurons or peripheral glial supporters within the brain, ultimately preceding degenerative insults.

Following brain injuries and diseases, growth factors such as NGF and brain-derived neurotropic factor (BDNF) play an integral role in the brain, notably in sustaining the repair of neurological damage, dominating neural regeneration, regulating neuroendocrine, as well as contributing to the maintenance of brain homeostasis. Alternating BBB permeability due to brain injury enhances brain BDNF depletion, and reductions in BDNF levels elicit neurodegeneration and mood disorders (Lesniak et al., 2021). Rats with spinal cord injury exhibited an under-regulated condition of BDNF expression for at least 1 week, negatively impairing the hippocampal plasticity in the brain (Fumagalli et al., 2009). Upon brain injuries and diseases, neurogenesis is demanded imperatively by neural tissues at the lesion site to compensate for the damage in neurons and glial cells. In this regard, BDNF is a key chemotactic/trophic factor that recruits newly generated neuroblasts into the vasculature and translocates them from a neural stem cell genesis locus, typically the subventricular zone (SVZ), to the lesion site for subsequent neurogenesis (Lin et al., 2021). Beneficial modulatory strategies of growth factors will most certainly facilitate the management of neurological disorders.

Esvald's lab analyzed dynamic BDNF manifestation and spatiotemporal expression of its receptors TrkB and p75NTR in developing mammalian neural and non-neural tissues. This research contributes to the understanding of BDNF regulation and signaling throughout neuronal development. It also reminds the relevance of BDNF in hippocampal memory and learning processes during the developmental phase of the brain (Esvald et al.).

Correspondingly, hippocampal BDNF constitutes a pivotal target in neurodegenerative diseases. Hashemi and Ahmadi employed rat model of memory impairment by kainic acid-induced hippocampal injury. Treatment with a natural ingredient, α-pinene, mitigated neuronal decay and reinstated signaling pathway of BDNF/TrkB/CREB in the hippocampus, so as to alleviate the memory impairment. Given that monoterpene essential oils have antioxidant, anti-inflammatory, and anti-apoptotic effects, natural compounds with these properties have the potential to be applied to the treatment of neurodegenerative diseases (Hashemi and Ahmadi).

As mentioned earlier, neurotrophins NGF and BDNF are principal growth factors; however their clinical therapeutic potential is limited due to their inferior pharmacokinetic properties. This necessitates the synthesis of small molecule prototypes that display high affinity for binding to the respective receptors TrkA and TrkB to achieve protective effects of NGF and BDNF. Narducci's team investigated the synthesis of two series of endogenous neurosteroid dehydroepiandrosterone (DHEA) derivatives, totaling 27 compounds, which have been identified as small-molecule selective agonists of TrkA and TrkB receptors as neurotrophin precursor analogs for therapeutic agents for neurodegenerative diseases (Narducci et al.).

Pathological states of the nervous system, whether injury or degeneration, may modify the high-affinity binding of BDNF to the receptor TrkB, which in turn leads to the development of neurocognitive disorders and cognitive decline. The pathogenesis of Alzheimer's disease and psychiatric disorders has been shown to correlate with downregulation of the BDNF/TrkB complex, and translational strategies to upregulate the BDNF/TrkB system will potentially contribute to the therapeutic treatment of these brain disorders. The above is described in the review article of Numakawa and Kajihara.

Finally, BDNF production can be induced by non-pharmacological strategies, such as physical exercise. The production of BDNF induced after physical exercise may originate from neuronal-dependent overexpression, increased cerebral blood flow, and humoral response. These three major mechanisms are discussed in the review article by Cefis et al.. As discussed in the review, crosstalk between the brain and systemic organs (e.g., liver, muscle) contributes to brain BDNF regulation, as well as modulation of neuroinflammation to combat degenerative brain diseases (Cefis et al.).

Taken together, these articles explore innovative technologies and reflect the latest advances in the field of neurological injuries and diseases. We enthusiastically hope that this Research Topic will inspire further innovative research and that researchers will advance scientific knowledge of neuromolecular basis and improve patient care.

## Author contributions

W-JC: Writing – original draft. W-MK: Writing – original draft. M-SL: Writing – review & editing.
